# Effect of amino-acid intake on physical conditions and skin state: a randomized, double-blind, placebo-controlled, crossover trial

**DOI:** 10.3164/jcbn.18-108

**Published:** 2019-05-24

**Authors:** Motoko Takaoka, Saki Okumura, Taizo Seki, Masaru Ohtani

**Affiliations:** 1 Department of Biosphere Sciences, School of Human Sciences, Kobe College, 4-1 Okadayama, Nishinomiya-shi, Hyogo 662-8506, Japan; 2 Groupwide Research and Development, Noevir Co., Ltd., C-333 R&D KSP, 3-2-1 Sakado, Takatsu-ku, Kawasaki, Kanagawa 213-0012, Japan; 3 Meiji University International Institute for Bio-Resource Research (MUIIBR), Kawasaki, Kanagawa 214-8571, Japan; 4 DAC Co., Ltd., 6-12-12 Ebara, Shinagawa-ku, Tokyo 142-0063, Japan

**Keywords:** amino acids, l-leucine, l-arginine, l-glutamine, body compositions/skin conditions

## Abstract

The objective of this study is to elucidate the effect of a supplement enriched with l-leucine, l-arginine, and l-glutamine on body compositions/skin conditions. Healthy young women (*n* = 29) were allocated to a group (*n* = 14) receiving an amino-acid supplement (600 mg l-leucine, 250 mg l-arginine, and 300 mg l-glutamine) and a placebo group (*n* = 15) receiving a supplement not-containing the amino acids. The amino-acid supplement and placebo were given twice/day for 6 weeks. After a wash-out (2 months) from the 1st test, the amino-acid group received the placebo and the placebo group the amino-acid supplement. The body compositions/skin conditions were measured 4 times (day 1 and weeks 2, 4, and 6) in each test. Percentage-change of muscle mass in the amino-acid group increased up to 4 weeks (*p* = 0.05) and was higher than that in the placebo group (*p* = 0.09). Skin texture estimated by the image processing of neck skin replica tended to increase in the amino-acid group at 6 weeks compared with that at 0 week, though there was no significant intergroup difference. In conclusion, the young adult women having no fitness habit showed the significant increase of the muscle amount and improvement tendency of the skin texture by the continuous intake of the amino-acid supplement.

## Introduction

In 2000 the World Health Organization (WHO) has defined the healthy life expectancy (HALE) at birth as the average number of years during which one can enjoy healthy life without any care, and clearly stated its significance in human life. Since then, many people in the world have come to recognize that expanding HALE is important together with expanding average life expectancy. Health risks/problems including osteoporosis, skin damage, perimenopausal symptoms, and presbyopia have increased with aging in any people. It has been, however, thought that we can prevent various diseases caused by aging by suppressing or delaying the decline of body functions and as the result extend both HALE and average life expectancy. This concept is called “anti-aging medicine” and we have thought that daily habits of eating, fitness, and sleep, and cosmetic cleaning are included as the important factors that are essential for anti-aging. Consciousness about health and anti-aging is high especially in middle-aged and elderly, while most women of broad generation tend to have common desires to become beautiful and highly interest in weight control. Thus, the number of population which habitually takes health supplements has increased to result in the expansion of a worldwide market of healthy foods whose main purpose is to attain the anti-aging, slim figure, health maintenance, beautiful skin, and the like. Mainstream of the past may need beauty cosmetics, but healthy supplements having effects from the inside of a body have attracted more attention recently. As a result, sales target of various beauty supplements includes all generations and the consumption of amino-acid supplements has been expanding in the supplement market worldwide; thus, the growth of the demand for such supplements is not temporally but continuously.^(1)^

The objective of this study is to elucidate the effect of a supplement enriched with l-leucine, l-arginine, and l-glutamine on body compositions/skin conditions, because physiological significance of these amino acids is quite important as described below. Therefore, the supplement used in this study was enriched with the three amino acids in addition to some vitamins.

The signature of the physical aging appears as the change in body compositions including the decrease of muscle mass and the increase of fat amount. The decrease of muscle mass will cause the drop of the physical capacity and the increase of risks such as falling. Muscle synthesis responding to assimilation of food taken decreases with aging probably due to the reduction of susceptibility in protein synthesis. At least a part of the reduction of susceptibility will be ascribed to the decrease of physiological concentration of l-leucine, which is one of branched chain amino acids (BCAAs).^(2)^ A study on aged rats confirms that l-leucine stimulated muscle protein synthesis.^(3)^ Pasiakos *et al.*^(4)^ describe that muscle protein synthesis was accelerated with a supplement enriched with l-leucine in convalescing person who had exercised and presume that the intake of l-leucine after exercise is effective for the promotion of synthesis of body proteins. Indeed, l-leucine affects a signal pathway involved in metabolic control.^(5)^ Metabolic syndrome is a worldwide social problem at present and a possibility that intake of l-leucine is effective in the prevention of overweight and diabetes was indicated.

l-Arginine is an essential amino acid for infants/children and under certain conditions for adults. When one suffers from an injury and an infectious disease, sufficient supply of l-arginine is desirable, because this amino acid is important in certain biological systems and is known to have a function to stimulate immune reactions through activation of T-cells.^(6)^ l-Arginine is metabolized *in vivo* to nitric oxide (NO) which has diverse physiological functions such as vasodilation, the improvement of exercise ability, the improvement of capacity of nutrition supplement to each organ, the suppression of accumulation of fatigue substances, and the improvement of antioxidative property.^(7,8)^ Increase of growth hormone was also observed in blood after intake of l-arginine supplement.^(9)^

l-Glutamine is an amino acid whose amount is the highest in human muscle and plasma accounting for approximately 60% of total free amino acids in a body^(10)^ and is the major source of energy in intestinal mucosal cells, fibroblasts, lymphocytes, and macrophages. Though this amino acid is not an essential amino acid, it is an indispensable ingredient for the normal growth of human culture cells.^(11)^ This amino acid is involved in the DNA synthesis as a precursor of nucleic acids and nucleotides.^(12)^ In addition, l-glutamate is the precursor of l-arginine which generates NO as described above^(13)^ and glutathione, the major biological antioxidant.^(14)^ l-Glutamate is supposed to have an anti-oxidation capacity and reported to resolve diarrhea and improve an intestinal barrier function after its oral administration in infants with diarrhea or malnutrition.^(15)^ l-Glutamine additionally participates in the immune function^(16)^ and is supposed to elevate the ability of anti-oxidation, organ protection, and immunity, and to support the metabolism, and the synthesis and degradation of proteins.^(17)^

There are many studies on the effects of amino acids including l-leucine, l-arginine, and l-glutamine on health. Each amino acid possesses specific functions described above, while combination of these amino acids is presumed to give different functions from those observed in the single amino acid. There are, however, few in-depth reports on the functions obtained by combining amino acids though we can access reports on the functions observed in the mixture of BCAAs. In addition, subjects in those studies were person with specific disease, aged people, or athletes though there are a few reports on the healthy young subjects.^(18,19)^ Based on these situation and backgrounds, we performed a clinical trial using healthy young Japanese women who have no fitness habits to make clear the function(s) caused by an amino acid-containing supplement enriched with the three amino acids, i.e., l-leucine, l-arginine, and l-glutamine. The trial is a placebo-controlled, double-blind, crossover study in which the subjects ingested the supplement enriched with the amino acids for a prescribed period, and cosmetic and health effects were examined by skin tests and observations of body composition change, respectively.

## Materials and Methods

### Trial registration

This clinical trial was registered with the University Hospital Medical Information Network (UMIN) on May 19, 2015 (Registration Number: UMIN000017615).

### Subjects

Twenty nine Japanese female students and staff aged 20 years or older in Kobe College from whom consent was obtained in written form in advance participated in this trial voluntarily. The subjects included in this trial did not suffer from any problematic diseases under treatment, not regularly take pharmaceutical products or supplements, and not smoke cigarettes. This study was performed in conformity with the purport of Helsinki Declaration (issued in 1964 and modified in 2008), and under the full consideration of medical ethics. The study was reviewed and approved by Ethical Review Board of Kobe College Graduate School of Human Sciences in 2015. All measurements were conducted in facilities in Kobe College. Even if a subject once agreed to participate in the study, it was decided that the subject was able to retire from the participation at any time including duration of the study without any disadvantage according to her free will.

### Study design

The study was conducted based on a placebo-controlled, double blind, randomized, cross-over design. Prior to implementation of the study, we explained the experiment contents orally and by using text to the participants. Table 1 shows baseline characteristics of subjects. During the study period, it was prohibited to ingest a new supplement containing amino acids and vitamins, to apply special cosmetics to the skin part to be observed, and to use cosmetic technique such as chemical peel. The skin moisture content in the neck of the participants was measured in advance and they were allocated in 2 groups based on the skin moisture content used as the assignment factor. The subjects and relevant practitioners were double-blinded. The consigned controller of this study was a staff of a clinical test support organization, IBEC Co., Ltd. (Osaka, Japan).

The controller assigned each subject into the 2 groups in the order of decreasing values of the skin moisture content perfectly randomly. To the 2 groups, 14 and 15 subjects each were assigned; the subjects (21.5 ± 2.1 years) in one group (AA group) took a supplement containing amino acids mixture of l-leucine, l-asparagine, and l-glutamine shown in Table 2, while those (22.3 ± 3.4 years) of another group (P group) took a placebo in which the amino acids in the above supplement were replaced with equivalent quantities of maltitol. In the study, the supplement and placebo were exchanged by the crossover method while setting a washout period for 2 months between the two experiments.

### Test supplements

The test supplement contained the following ingredients (total amount: 2,500 mg) in a bag (Table 2): totaling 1,150 mg of amino acids consisting of l-leucine 600 mg, l-arginine 250 mg, and l-glutamine 300 mg, citric acid (acidifier), maltitol and sucralose (sweetener) as excipients, and multivitamins (vitamins C, B_1_, B_2_, B_6_, B_12_, A, E, D, niacin, pantothenic acid, folic acid). The placebo sample was prepared by substituting only the amino acids by maltitol in the supplement without any alteration of other ingredients (Table 2).

### Experimental design

The subjects took 1 package each of the test supplement or placebo after dinner and before bedtime (2 packages in a day) for 6 weeks. The measurement was conducted 4 times during the test at 0 week (before ingestion), 2, 4, and 6 weeks. After a washout period for 2 months from the end of the 1st test period, the test supplement containing amino acids was replaced with the placebo and vice versa in the 2nd test period. The first test was performed from May 26 to July 9 in 2015, and the second one in which the groups were rearranged from October 21 to December 17 in 2015.

The subjects were instructed to avoid overeating, excessive alcohol consumption, excessive exercise, and poor sleep during the test as to living habits. A meal frequency was examined before and after the study to confirm the eating style of the subjects during the test.

### Measurements

After the subjects changed into prescribed clothes (sweat suit of top and bottom set), a body component was measured with a body composition analyzer (Jawon Medical Co., Ltd., Daejeon, South Korea). After measurement of the body composition, the subjects washed the inner side of the forearm and the neck lightly, measuring points, using a designated detergent, and were allowed to sit for 20 min for the acclimation to the measurement environment. A skin test was conducted in a temperature/humidity-controlled room (temperature: 21.0 ± 1.0°C, humidity: 55.0 ± 10.0%). The measurement points for skin moisture content were at the inner side of the forearm determined using an exclusive ruler and at the fixed point of the neck where the line taken down from the joint of an ear and the line pulled to an Adam’s apple crossed vertically. Moisture content of the skin was measured with a skin surface hygrometer Skicon-200EX (IBS Co., Ltd., Hamamatsu, Japan). Transepidermal water loss (TEWL) was measured with Tewameter^®^ TM300 (Courage + Khazaka electronic GmbH, Cologne, Germany). Skin color (redness and yellowness) at each specified region at the neck was measured 5 times to calculate the average values with a colorimeter (CM2600, Konica Minolta Co., Ltd., Tokyo, Japan). Skin brightness was measured by a specular component excluded (SCE) method to correctly observe the targeted position by eliminating specular reflection light. The skin viscoelastic character at the inner side of the forearm was measured by using a skin viscoelasticity measuring instrument (Cutometer^®^ dual MPA 580, Courage + Khazaka electronic GmbH, Köln, Germany). R2 (skin elasticity: recovery ratio of skin length), R6 (skin sagging: ratio of viscosity and elasticity when elongated), and R7 (skin firmness: ratio of elasticity during constriction) were used as the main parameters of Cutometer to assess skin elasticity and to measure elasticity of the upper skin layer using negative pressure which deforms the skin mechanically. Replicas of the skin surface of the designated regions at the inner side of the forearm and the neck were collected using silicone impression material kit, ASB-01 (Asahi Biomed Co., Ltd., Yokohama, Japan).

### Statistics

Experimental data are represented as the means ± SD. The intergroup and intragroup differences of mean values were interpreted by Student’s paired *t* test. Two-way (group × time) repeated-measures ANOVA was used to determine the significance of differences between and within the groups. Differences were considered statistically significant when *p* value is less than 0.05 (*p*<0.05). All data were analyzed by using SPSS (ver. 18.0, 2006; SPSS Inc., Chicago, IL).

## Results

**Fig. 1:**
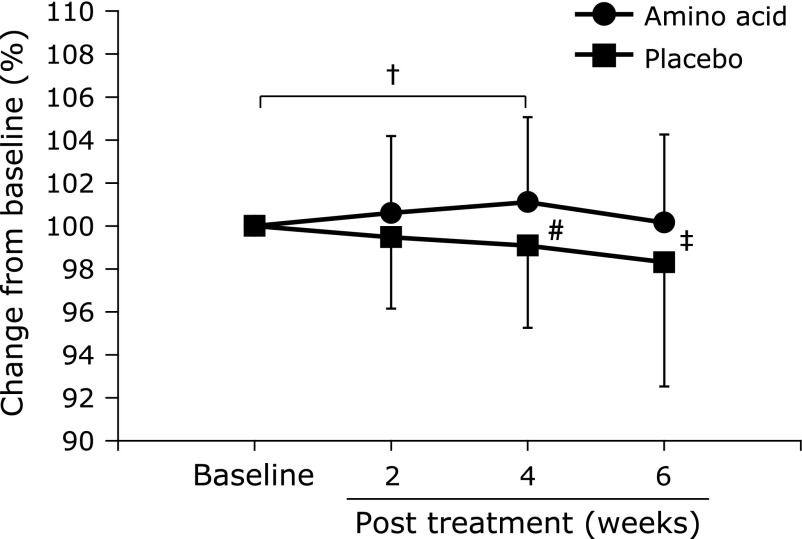
Percentage change in muscle mass from baseline in AA and P groups. P group: placebo group, AA group: group receiving an amino acid-containing supplement. ^†^*p*<0.1 vs baseline (100%), ^#^*p*<0.05, ^‡^*p*<0.1 vs P group, means ± SD. Results of two-way ANOVA: Treatment: F(1, 196) = 7.8, *p* = 0.006; week: F(3, 196) = 0.6, *p* = 0.6; interaction at week x of ingestion: F(3, 196) = 1.3, *p* = 0.3.

Table 3 shows the change of the body weight and body compositions of the subjects in the AA and P groups. The weight tended to decrease from the starting point (0 week) in both of the AA and the P groups without significant intergroup and intragroup differences. No significant difference was observed in the amount of body fat and the value of % of body in the P group throughout the testing period, while those in the AA group tended to decrease up to 4 weeks at which *p* value was <0.1 (vs 0 week). The % change of muscle mass from the baseline increased in the AA group, but decreased in the P group; as a result the % change in the AA group was significantly higher than that in the P group at 4 weeks (Fig. 1). Muscle mass was likely to increase by the amino acid supplementation. Two-way ANOVA showed no significant time effects, but a tendency of group effect was observed for the % change of muscle mass amount [*p* = 0.06, F(1, 196) = 7.8].

The skin moisture content, one of the indices to evaluate the skin conditions, exhibited a tendency of increase from the baseline in the neck of subjects in both groups of AA and P (Table 4). The skin moisture content at the forearm significantly increased at 2 weeks in the AA group and 3 weeks in the P group (data not shown). In addition, in comparison with the starting time, significant increase of the skin moisture content at the neck was observed at 4 weeks in the AA group (*p*<0.05) and at 2 and 6 weeks in the P group (*p*<0.01, respectively). The TEWL tended to decrease with time in the skin of the forearm and the neck in both groups; the decrease of TEWL in the forearm skin was significant at 6 weeks in the P group (data not shown). Table 4 demonstrates that the TEWL in the skin of the neck significantly decreased from the baseline at 4 and 6 weeks in the AA group (*p*<0.05 and *p*<0.01, respectively) and at 2, 4, and 6 weeks in the P group (*p*<0.01, *p*<0.05, and *p*<0.01, respectively). Neither skin moisture nor TEWL in the skin of the neck were significantly different between the AA group and the P group (Table 4). Skin elasticity was measured with a cutometer. Indices of skin elasticity used for the analysis were R2 indicating viscoelasticity of the skin, R6 showing the degree of the slack, and R7 indicating the degree of skin tension. As a result, none of the parameters examined showed significant intergroup difference in the skin of the neck in the AA group and the P group (Table 5).

Next, the skin color was examined using a colorimeter according to the SCE procedure (Table 5). In the skin at the inner side of the forearm and the neck in either the AA group or P group, the brightness increased and the increase at 6 weeks was statistically significant in the P group (*p*<0.01). Brightness of the skin at the neck in the P group was higher than that in the AA group at 6 weeks without significant difference (Table 5). Redness of the skin color tended to increase in the P group, while yellowness of the skin decreased significantly in the AA group at 6 weeks (*p* = 0.05). The slight skin redness in the AA group was slight, but significantly higher than that in the P group at 6 weeks.

A replica of the skin was picked followed by estimating skin texture by an image processing of the skin replica. Uniformity of the skin texture is estimated by the ratio of volume rate of the skin texture to the rate of whole skin volume. The higher the ratio became, the higher the uniform texture achieved.

**Fig. 2:**
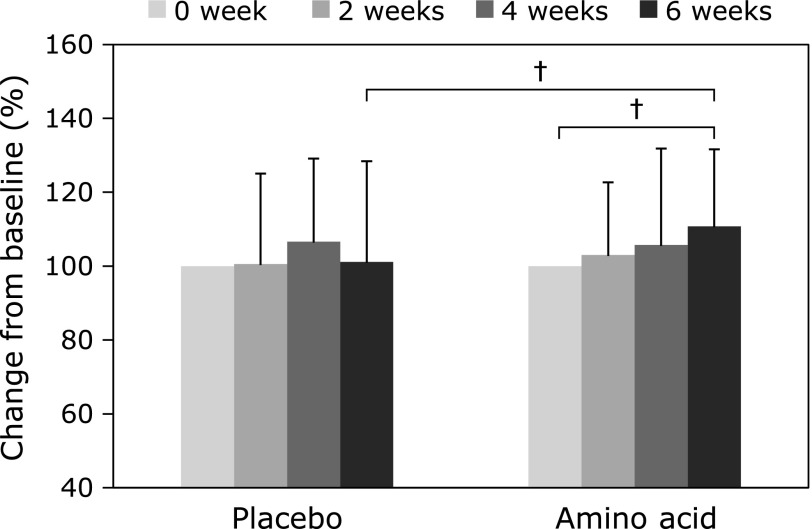
Changes in the skin texture of neck following ingestion of the amino acid-containing supplement and the placebo. Means ± SD, ^†^*p*<0.1 compared to Placebo group and 0 week.

The % change of the skin texture of the neck was observed to significantly increase in the AA group at 6 weeks (*p* = 0.03), while the forearm skin texture did not change in both AA and P groups (Fig. 2).

## Discussion

In this study, we examined the changes in the body constitutions and the skin conditions in healthy adult women having no habitual exercise during and after taking either supplement not containing amino acids (P group) or that containing l-leucine, l-arginine, and l-glutamine (AA group) for 6 weeks. In the study, the body constitutions and the texture of skin were improved in the subjects belonging to the AA group though the change of weight was similar in both AA and P groups. In the AA group, body fat tended to decrease, while body muscle amount increased up to 4 weeks in comparison with the starting time. Thus, there observed a tendency of intergroup difference in the increase of the body muscle amount which was higher in the subjects in the AA group than those in the P group. Based on these results, we concluded that the continuous intake of the amino acid supplement containing l-leucine, l-arginine, and l-glutamine for at least 6 weeks tended to increased the muscle amount of the young adult women. Paddon-Jones *et al.*^(20)^ report similar result in which the intake of essential amino acids stimulated muscle protein synthesis in the young and elderly people.

Hereafter we will discuss the respective effects of the three amino acids on the present results.

The followings have been elucidated as the role of l-leucine in several studies hitherto conducted: (1) l-leucine is an amino acid indispensable for protein synthesis ^(21)^ and a potent activator in the protein synthesis in white adipose fat-cells via a similar pathway,^(22)^ (2) the carbon skeleton of l-leucine is utilized for the production of ATP, and (3) l-leucine is an activator of the mechanistic target of rapamycin (mTOR) whose signaling is important *in vivo* to control metabolism and the multiply, growth, and survival of cells. Based on these results, the effects of intake of l-leucine have been extensively studied in the various situations such as aging, muscle disorders, protein/energy deprivation, overweight, and genuine diabetes;^(23,24)^ overall these results indicating that l-leucine is most likely to affect both muscle mass and fat mass in the various physical situations. Pasiakos *et al.*^(4)^ performed a clinical study in which the following two groups were compared after physical exercise; one group took essential amino acid supplement and another group l-leucine-enriched essential amino acid supplement. The latter supplement elevated the plasma concentration of l-leucine followed by activation of the muscle cells and muscle protein synthesis during convalescence of fatigue in certain types of exercises requiring stamina.^(4)^ In our study, similar results were obtained though the enrolled subjects did not perform habitual exercise. Katsanos *et al.*^(2)^ report that the promotion of a muscle protein synthesis by ingestion of l-leucine was different between young and elderly person; in young adults the enhancement of muscle protein synthesis by l-leucine occurred regardless of its ingested amount, whereas in elderly adults high concentration of l-leucine was required for the enhancement. There is a report that the intake of l-leucine did not change the body protein amount in aging model mice and elderly people, though l-leucine stimulated body proteosynthesis.^(25,26)^ Our result found in the healthy young women taking 1,200 mg of l-leucine per day in the AA group was well compatible with the above results. Thus, the present results observed in the young adult may not be accepted in elderly people.

There are several reports describing the effects of l-leucine on the body fat reduction,^(27)^ the increase of energy expense in an obese model mouse,^(28)^ and the increase of the adipose tissue by 25% without increasing food consumption in a young rat.^(29)^ However, since a survey using a simplified-food frequency questionnaire implemented during the experimental period of this study confirms no significant change in the amount and contents of diets taken by the subjects (data not shown), the change of body compositions in this study was supposed not to attribute to the change of dietary amounts and contents. When l-leucine was orally taken as a supplement, no change was observed in the intake of the diet,^(5)^ though there is a report describing that direct injection of l-leucine into the brain caused the restriction of intake of food.^(30)^ In conclusion, l-leucine was likely to increase the muscle amount and decrease the body fat without significant changes in the weight and dietary habits.

l-Glutamine has the following effects or rolls *in vivo*: (1) control of manifestation of many genes related to metabolism, signal transduction, cell defense, and repair,^(31)^ (2) activation of intracellular signaling pathways, (3) an energy source of cells of the immunity and digestive systems with rapid turnover, (4) a precursor of glutathione, an important anti-oxidant *in vivo*,^(32)^ and (5) control and stimulation of the turnover of proteosynthesis. Furthermore, restraint of proteolysis is reported in culture cells of the mouse skeletal muscle.^(33)^ Therefore, other than l-leucine, l-glutamine may be involved in at least a part of the increase of the muscle amount through proteosynthesis in the AA group. Many researchers have tried to verify the effect of l-glutamine from the viewpoints of medical nutrition^(17,34)^ and elucidated that this amino acid is important in maintaining functional status of the bowel and good physical conditions, and acts as an energy source of cells in the immune and digestive systems. Actually, oral administration of 30 g of l-glutamine to obese and overweight adults for 14 days resulted in the decrease of the ratio of *Firmicutes* to *Bacteroides*, a good biomarker for obesity.^(35)^ Though the amount of l-glutamine taken in this study was much smaller and 1,200 mg/day, there was a possibility that the intake of l-glutamate changed the composition of intestinal flora followed by reduction of the body fat. A natural moisturizing factor (NMF) consisting of various components including amino acids holds the key for the moisture content of the skin by maintaining water in the corneocytes, and thereby moisturization of the stratum corneum (SC) of the epidermal keratinocyte is achieved. Thus, the decrease of NMF is accompanied by the decrease of moisture in the corneocytes, disturbance of the arrangement of the corneocytes, formation of the dried and flaking skin, and aggravation of skin conditions. Main components of NMF are amino acids (approximately 40%) formed by degradation of filaggrin (filament aggregating protein). In our study, TEWL (indicator of decrease of moisturization) tended to decrease by the intake of amino acids in the skin of the forearm and the neck in both AA and P groups. The decrease of TEWL indicated the improvement of the barrier function of the SC and skin conditions are closely related to gastrointestinal functions and status. Constipated state will increase corruption products in the gut which in turn aggravate skin functions and conditions.^(36)^ l-Glutamine will maintain the gut in desirable conditions and is likely to have a function to maintain the homeostasis of organs including the gut and the skin. These results and observations are consistent with the improvement of skin texture (smoothness) at the crista and sulcus cutis examined by dermoscopic images in our study. l-Glutamine by which the environment in the gut was arranged and nutritional absorption efficiency was elevated is potentially associated with the skin texture improvement. In addition, l-glutamine is converted to glutathione whose strong antioxidant property is probably contribute to the improvement of skin conditions. Contrary to our expectation, however, the improvement extent of the skin conditions including the moisture content, TEWL, and viscoelasticity was approximately the same in the AA and P groups. This result may be explained by the effect of multi vitamins as suggested by Boyera *et al.*^(37)^

l-Arginine plays the central role in several biological systems including immune functions^(6)^ and the intake of l-arginine-enriched supplement enhanced the potency of self-antitumor immunity, prolonged survival time, and inhibited the growth of tumor in a cancer model mouse by inhibiting the number of myeloid-derived suppressor cells.^(38)^ l-Arginine is a substrate of both arginase and nitric oxide synthase (NOS) and is converted to urea and l-ornithine by arginase and to NO by NOS. l-Ornithine is a precursor of l-proline and important in the growth and restoration of cells. l-Proline biosynthesized from l-arginine via l-ornithine is the main constituent amino acid of collagen proteins. There is a possibility that collagen regeneration in the dermis is promoted by l-proline converted from ingested l-arginine, and thereby viscoelasticity of the skin improved. NO expands the blood vessel and improves blood stream.^(7)^ In addition, a study on the effects of intake of l-arginine supplement in rats shows a significant increase of NO level in blood, a decline of an oxidation stress, and a prolongation of an exercise time.^(7)^ Shan *et al.*^(8)^ demonstrate that l-arginine supplementation caused the promotion of the bioavailability of NO and the elevation of the anti-oxidative potency. The present study has confirmed the improvement of skin texture and skin viscoelasticity in the AA group. This observation can be explained by the follows: (1) the enhancement of NMF production in the epidermis and collagen regeneration in the dermis caused by the increase of blood stream by NO produced from l-arginine and the increase of supply of nutritious food matters, (2) the restraint of an oxidation stress of cells caused by anti-oxidative activity of l-arginine, and (3) the promotion of collagen regeneration by l-proline biotransformed from l-arginine.

The results described above indicate that the intake of the supplement containing l-leucine, l-glutamine, and l-arginine for 6 weeks changed the body constitutions such as the increase of the muscle mass and the decrease of the fat amount. The highest muscle amount was achieved at 4 weeks of the intake with a statistically significant difference in comparison with that of the P group, followed by a slight reduction at 6 weeks, though the reason(s) for this remain unclear at present. The improvement of skin texture was attained in the AA group. These findings were attributable to the additive effects produced by the combination of the three amino acids. Astaxanthin has high antioxidant and anti-inflammatory activities, and long-term astaxanthin supplementation suppresses the deterioration of wrinkles, moisture content, and interleukin-1α levels generated by environmental factors, such as UV and dryness, in a clinical study.^(39)^ In addition, the oral administration of a dried whole egg powder of farmed soft-shelled turtles, *Pelodiscus sinesis*, containing various proteins and amino acids inhibits the formation of advanced glycation end-products in the serum and skin of diabetic rats.^(40)^ Based on these reports, it is expected that the supplements used in this study can be combined with ingredients with high anti-glycation and anti-oxidant properties to develop more functional supplements to the skin.

Although this study provides the comprehensive and in-depth insight into the effect of the combination of l-leucine, l-glutamine, and l-arginine on the body components and skin conditions in younger women, several limitations have to be discussed. First, there are no placebo arms for the single amino acid, which enable to clearly demonstrate the role of the amino-acid combination. Second, since the sample size was small and only younger women were enrolled, generalizability of the data needs to be regarded with caution.

In conclusion this study shows that the consumption of the amino acid mixture affected the body compositions and the skin conditions of the healthy young women, and we can expect novel utilities of amino acid supplement based on these effects.

## Figures and Tables

**Table 1:** Baseline characteristics of subjects

Characteristic	*n* = 29
Age (year)	22.2 ± 3.4
Hight (m)	1.59 ± 0.048
Body mass index (kg/m^2^)	20.27 ± 2.15
Body weight (kg)	51.5 ± 6.5
Fat (kg)	12.1 ± 3.2
Mineral (kg)	3.0 ± 0.3
Muscle mass (kg)	36.4 ± 3.6

Value are means ± SD.

**Table 2:** Amino-acid and vitamin compositions per pack for clinical trial and DRI

Materials	Placebo group		Experimental group			Dietary reference intakes (DRI) for Japanese females aged 18–29 years			
	Amount per pack (mg)	Amount per day (mg)	Amount per pack (mg)	Amount per day (mg)		Estimated average requirement (EAR)	Adequate intake (AI)	Tolerable upper intake level (UL)	Units
l-Leucine	0	0	600	1200		—	—	—	
l-Arginine	0	0	250	500		—	—	—	
l-Glutamine	0	0	300	600		—	—	—	
Vitamin A	0.45	0.9	0.45	0.9		0.45	—	2.7	mgRAE/day
Vitamin B1	1.4	2.8	1.4	2.8		0.9	—	—	mg/day
Vitamin B2	1.6	3.2	1.6	3.2		1	—	—	mg/day
Vitamin B6	2	4	2	4		1	—	45	mg/day
Vitamin B12	0.004	0.008	0.004	0.008		0.002	—	—	mg/day
Niacinamide	11	22	11	22		9	—	250	mg/day
Folic acid	0.4	0.8	0.4	0.8		0.2	—	0.9	mg/day
Pantothenic acid	5.5	11	5.5	11		—	4	—	mg/day
Vitamin C	170	340	170	340		85	—	—	mg/day
Vitamin D	0.005	0.01	0.005	0.01		—	0.0055	0.1	mg/day
Vitamin E	8	16	8	16		—	6	650	mg/day

**Table 3:** Weight and body compositions at 0, 2, 4, and 6 weeks after initiation of daily ingestion of supplement containing amino acids or placebo

	Placebo group (*n* = 29)					Experimental group (*n* = 29)			
	0 week	2 weeks	4 weeks	6 weeks		0 week	2 weeks	4 weeks	6 weeks
Weight (kg)	51.6 ± 6.6	51.5 ± 6.5	51.4 ± 6.4	51.3 ± 6.3		51.5 ± 6.1	51.5 ± 6.0	51.4 ± 6.1	51.3 ± 6.0
Water (kg)	14.6 ± 3.1	14.5 ± 3.1	14.4 ± 3.0	14.4 ± 3.1		14.4 ± 2.9	14.5 ± 2.8	14.5 ± 2.8	14.4 ± 2.8
Fat (kg)	12.8 ± 4.0	12.7 ± 3.8	12.7 ± 3.7	12.6 ± 3.4		13.0 ± 3.5	12.8 ± 3.4	12.6 ± 3.5	12.6 ± 3.5
% of fat (%)	24.3 ± 5.2	24.3 ± 5.0	24.3 ± 4.6	24.2 ± 4.3		24.8 ± 4.5	24.5 ± 4.3	24.1 ± 4.5^†^	24.2 ± 4.5
Muscle mass (kg)	35.9 ± 3.3	35.8 ± 3.3	35.7 ± 3.0	35.8 ± 3.3		35.5 ± 3.1	35.7 ± 3.0	35.8 ± 3.1	35.7 ± 3.0
% of muscle mass (%)	69.9 ± 5.1	70.0 ± 5.0	69.9 ± 4.6	70.0 ± 4.2		69.4 ± 4.5	69.8 ± 4.2	70.4 ± 5.3^†^	70.1 ± 4.5

All values are means ± SD. ^†^*p*<0.1 vs 0 week.

**Table 4:** Changes in skin moisture content, TEWL, and skin texture of the neck after initiation of ingestion of amino acid-containing supplement and placebo

	Unit	Placebo group (*n* = 29)					Experimental group (*n* = 29)			
		0 week	2 weeks	4 weeks	6 weeks		0 week	2 weeks	4 weeks	6 weeks
Skin water content hydration	au	54.8 ± 12.5	62.4 ± 12.5**	57.1 ± 8.7	60.3 ± 10.2**		54.4 ± 9.1	54.7 ± 10.8	59.2 ± 10.4*	54.5 ± 8.6
Transepidermal water loss (TEWL)	g/(m^2^·h)	9.0 ± 5.0	7.5 ± 3.6**	7.5 ± 2.5*	6.7 ± 2.3**		8.3 ± 3.4	8.2 ± 3.6	7.4 ± 2.6*	6.6 ± 2.0**
Skin texture	—	0.54 ± 0.14	0.53 ± 0.16	0.54 ± 0.16	0.52 ± 0.16		0.49 ± 0.13	0.51 ± 0.15	0.51 ± 0.15	0.54 ± 0.17**

All values are means ± SD. *p<0.05, **p<0.01 vs 0 week.

**Table 5:** Changes in skin color and skin elasticity on neck skin with time following ingestion of amino asids and the placebo

		Unit	Placebo group (*n* = 29)			Experimental group (*n* = 29)	
			0 week	6 weeks		0 week	6 weeks
Skin color analysis by specular component included (SCI) method	Lightness (L*)	—	61.7 ± 4.1	63.4 ± 2.6**		62.1 ± 3.0	62.8 ± 3.4
	Redness (a*)	—	5.2 ± 1.1	5.4 ± 1.1		5.4 ± 0.9	5.4 ± 1.2
	Yellowness (b*)	—	20.0 ± 2.0	19.9 ± 2.0		19.8 ± 1.7	19.4 ± 2.0*

Skin elasticity of the upper skin layer	Gross elasticity (R2)	—	0.89 ± 0.05	0.91 ± 0.04*		0.89 ± 0.05	0.90 ± 0.04^†^
	Portion of the visco-elasticity of the curve (R6)	—	0.26 ± 0.09	0.28 ± 0.06		0.28 ± 0.10	0.31 ± 0.11
	Portion of the elasticity compared to the complete curve (R7)	—	0.72 ± 0.10	0.69 ± 0.12		0.70 ± 0.12	0.70 ± 0.09

All values are means ± SD. ^†^*p*<0.1, *p<0.05, **p<0.01 vs 0 week.
